# Adenomyoepithelioma of the breast with prominent cystic changes: a case report

**DOI:** 10.1186/s12905-021-01432-z

**Published:** 2021-08-04

**Authors:** Feng Chen, Hengping Wu, Yujian Liu, Minli Lv, Jianquan Zhong

**Affiliations:** Department of Radiology, Zigong First People’s Hospital, Zigong, 643000 Sichuan People’s Republic of China

**Keywords:** Adenomyoepithelioma, Breast, Cystic

## Abstract

**Background:**

Adenomyoepithelioma (AME) of the breast is a rare subtype of breast tumor. Most of AMEs reported are solid, however, cystic or prominent cystic changes are extremely rare.

**Case presentation:**

A 51-year-old woman presented a lump in the upper outer quadrant of right breast, and it was accompanied by continuous breast pain and bilateral axillary itching for more than 2 months. There were no other symptoms found. Preoperative mammography and ultrasound examination were performed. Mammography showed a noncalcified lobulated mass, and it was considered to be a benign cyst with septum on ultrasound, but ductal carcinoma of breast, adenoid cystic carcinoma could not be excluded. At first, AME was not considered preoperatively, because the imaging features of this rare tumor may vary widely, which may result in an incorrect diagnosis. But eventually, AME was diagnosed by postoperative pathology and immunohistochemistry.

**Conclusion:**

We herein present a rare case of breast AME with prominent cystic changes. AME has no-specific imaging features, but the benign or malignant nature of the lesion might be suspected on imaging.

## Background

Adenomyoepithelioma (AME) of the breast was first described by Hamperl in 1970 [1]. According to the World Health Organization classification of breast tumors in 2012, AME was classified as epithelial-myoepithelial lesions [2]. AME is generally a benign breast tumor displaying proliferation of epithelial and myoepithelial cells, mainly myoepithelial hyperplasia. Tavasoli [3] subdivided AMEs into three variants, arranged in spindle cell type, tubules, and lobules. The diagnosis of AME was established by histopathology and immunohistochemistry. Most of AMEs showed solid mass rather than cystic mass [4], cystic or prominent cystic changes is extremely rare, in spite of a few may contain small cystic areas [5].

## Case presentation

**Fig. 1:**
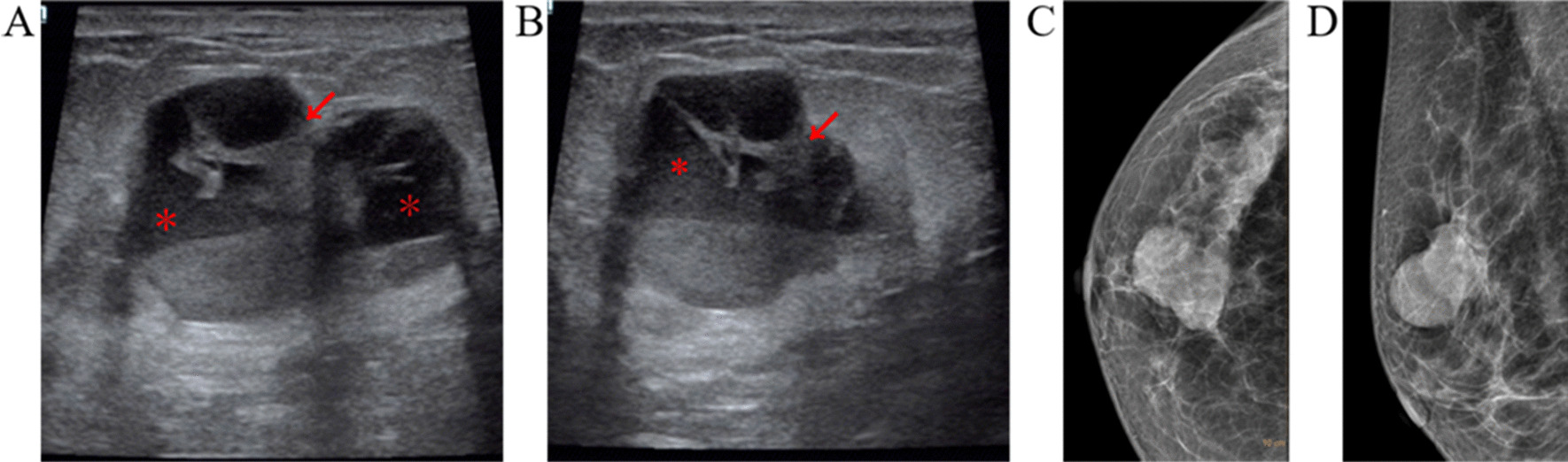
**a**, **b** Ultrasonogram revealed no echogenic (red *) and well circumscribed mass with solid striped septum (red ↙), weak echogenic deposition in the dorsal side. **c**, **d** On the CC and MLO mammograms showed lobulated mass without calcification

**Fig. 2:**
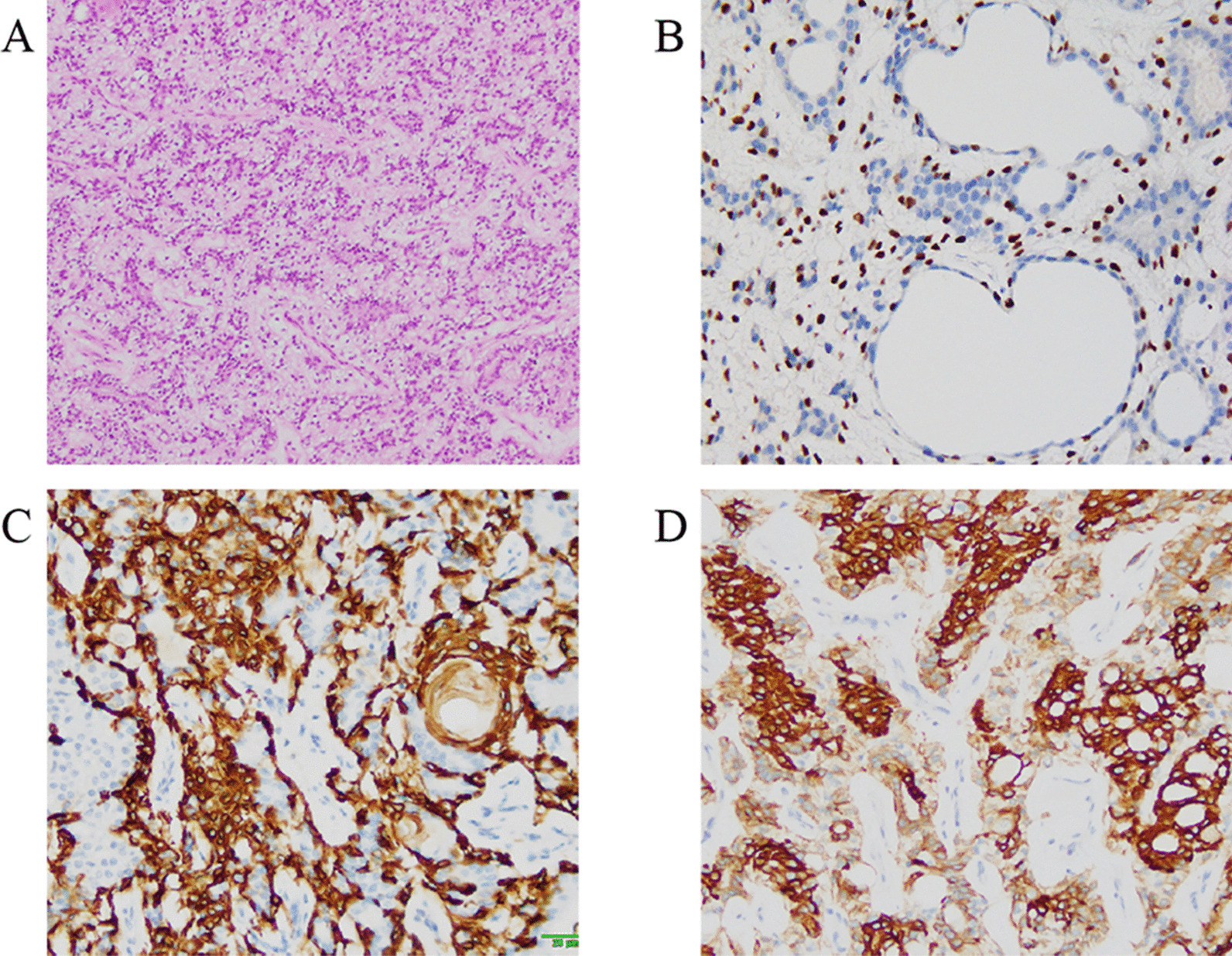
**a** Photomicrograph of histologic specimen showed that the tumor was biphasic appearance, with inner layer of epithelial cells and outer layer of myoepithelial cells (HE, ×200). **b** Nuclear P63 staining in the myoepithelial component confirmed the differentiation of the myoepithelium (streptavidin-perosidase, ×200). **c** CK5/6 highlights proliferating myoepithelial cells (streptavidin-perosidase, ×200). **d** Epithelial cells were very strongly positive for CK7 (streptavidin-perosidase, ×200)

A 51-year-old woman presented a lump in the upper outer quadrant of right breast, and it was accompanied by continuous breast pain and bilateral axillary itching for more than 2 months. There were no other symptoms found. Previously she didn't undergo any breast cancer screening, and had no family history of breast cancer. Physical examination revealed an irregular mass by palpation where was 3.5 cm away from the nipple, about 11 o 'clock in the right breast, with hard texture, clear boundary, mobility and mild tenderness. Doppler ultrasound (Fig. 1a, b) showed no echogenic and irregular mass in the right breast, with solid striped septum, and weak echogenic deposition in the dorsal side, and the mass was well circumscribed. There was no blood flow signal in the lesion. Mammography (Fig. 1c, d) showed a lobed mass with slightly higher density in the right breast, smooth edge, partially concealed boundary, and there was no calcification in the mass. It was classified as category 4A by BI-RADS assessment. It was considered to be a benign cyst with septum on ultrasound, but ductal carcinoma of breast, adenoid cystic carcinoma could not be excluded. We suggested the patient undergo MRI examination, but the patient refused. Since breast cancer could not be excluded, the patient was urged for a partial mastectomy. Postoperative general features: A 3.5 cm × 3 cm × 2.2 cm mass was observed at 11 o 'clock of the right breast, 3.0 cm from the nipple, with an unclear boundary and enveloped, also a 2.8 cm maximum diameter cyst was observed on the section, and a 2.2 cm × 1.8 cm × 1.5 cm gray-white neoplasm was observed inside the cyst. Micrographs of histological specimens with HE staining (Fig. 2a) showed the mass was biphasic appearance.The outer myoepithelial cells were single or multilaye, the cytoplasm was clear and the nuclei was not atypia. The inner layer were glandular epithelial cells, arranged in an adenoid structure, with abundant cytoplasm and no nuclear atypia. And immunohistochemistry (Fig. 2b–d) showed that: CK 5/6 (+, myoepithelial), P63 (+), calponin (+, myoepithelial), CK (+), CK7 (+, epithelial), L-CK (+, epithelial), SMA (−), H-caldesmon (−), so it was diagnosed as AME. Because of the pathology and immunohistochemistry showed the tumor was well differentiated, she did not receice any further treatment after the surgery.

## Discussion and conclusions

Breast AMEs are rare neoplasms. They have been described in patients ranging in age from 22 to 93 years [6], although most of them were elderly women, male cases were also reported [7]. AMEs are generally benign neoplasms, although a small number of malignant lesions have been reported in the literature, either the epithelial or myoepithelial component may undergo malignant transformation [8]. Some papers have concluded that AMEs over 2 cm should be treated as malignant [9]. Most of AMEs were solid, prominent cystic features of this tumor was extremely rare, in spite of rare minute cysts were described in a few cases of AMEs. A review of the literature indicates that only one case of cystic AME has been reported [10]. In our case, the patient had a large lesion with persistent breast pain. Although the lesions had prominent cystic changes, the boundary of some lesions was unclear, and she did not receive any further treatment after the surgery. It is important for the surgeon to achieve a clear margin when removing the tumor, because local recurrence or even malignant transformation can happen. So the patient still needed to be followed up to observe if malignant transformation occur after local lesion resection. Up to now, the patient has been followed up for half a year without recurrence or discomfort, and re-examination of ultrasound showed no abnormality. The radiological findings of breast AME are nonspecific. On ultrasound, AME typically presents as a solid, hypoechoic, small, irregular, or oval mass, often accompanied by posterior acoustic enhancement. Peripheral vascular enlargement has some features, and the mass may have catheter dilation [11, 12]. Mammography is usually characterized by a noncalcified ovoid or lobulated mass with smooth margins [13, 14]. MRI can provide additional information on the morphological and haemodynamic characteristics. On MRI, benign AMEs manifest as homogeneous signal on different sequences with Type I or II enhancement curves, while malignant AMEs presented as irregular and coarse-edged masses with type III enhanced curve [12, 15]. In our case, ultrasonography showed a lobulated mass with prominent cystic changes in the right breast, which was significantly different from the stereotypical AME images. Mammography shows a noncalcified lobulated mass, which is not significantly different from the common AME imaging findings due to the poor performance of the cystic changes on mammography. The prominent cystic features of the present tumor was easily misdiagnosed, so it’s needed to be distinguished from ductal carcinoma of breast, adenoid cystic carcinoma, lobulated tumor, cyst of galactostasia, metaplasia carcinoma, etc. The imaging findings of these tumors did not differ significantly.

AME of breast is a rare and mostly benign tumor. AME has no-specific imaging features, but the benign or malignant nature of the lesion might be suspected on imaging.

## Data Availability

Data sharing is not applicable to this article as no datasets were generated or analysed during the current study.

## References

[1] Hamperl H (1970). The myothelia (myoepithelial cells). Normal state; regressive changes; hyperplasia; tumors. Curr Top Pathol.

[2] Lakhani SR, Schnitt SJ, Tan PH, Vijver MJ. WHO Classification of Tumors of the Breast. World Health Organization Classification of Tumours. 2012.

[3] Tavassoli FA (1991). Myoepithelial lesions of the breast: myoepitheliosis, adenomyoepithelioma, and myoepithelial carcinoma. Am J Surg Pathol.

[4] Rosen PP (1987). Adenomyoepithelioma of the breast. Hum Pathol.

[5] Kim MJ, Kim CS, Ju MJ, Park YS (2019). Malignant adenomyoepithelioma of the breast: a rare case report. Int J Surg Case Rep.

[6] Antonelli MS, Mallel G, Pecoraro A, Vitale V, Maggi S, Lombardi A, Stanzani G, Amanti C. Adenomyoepithelioma of the breast: case report and literature review. G Chir. 2018;39:255–7. 30039795

[7] Gafton B, Scripcariu V, Prutianu I, Alexa-Stratulat T, Terinte C, Nicolau A, Moisiuc D, Radu I (2019). Challenges in management of male breast adenomioepithelioma with malignant behavior: case report. Medicine (Baltimore).

[8] Kakkar A, Jangra K, Kumar N, Sharma MC, Mathur SR, Deo SV (2019). Epithelial-myoepithelial carcinoma of the breast: a rare type of malignant adenomyoepithelioma. Breast J.

[9] Korolczuk A, Amarowicz M, Bąk K, Korobowicz E, Koncewicz T (2016). Adenomyoepithelioma of the breast with late pulmonary metastases—case report and review of the literature. J Cardiothorac Surg.

[10] Papaevangelou A, Pougouras I, Liapi G, Pierrakakis S, Tibishrani M, Setakis N (2004). Cystic adenomyoepithelioma of the breast. Breast.

[11] Lee JH, Kim SH, Kang BJ, Lee AW, Song BJ (2010). Ultrasonographic features of benign adenomyoepithelioma of the breast. Korean J Radiol.

[12] Park YM, Park JS, Jung HS, Yoon HK, Yang WT (2013). Imaging features of benign adenomyoepithelioma of the breast. J Clin Ultrasound.

[13] Adejolu M, Wu Y, Santiago L, Yang WT (2011). Adenomyoepithelial tumors of the breast: imaging findings with histopathologic correlation. AJR Am J Roentgenol.

[14] Moritz AW, Wiedenhoefer JF, Profit AP, Jagirdar J (2016). Breast adenomyoepithelioma and adenomyoepithelioma with carcinoma (malignant adenomyoepithelioma) with associated breast malignancies: a case series emphasizing histologic, radiologic, and clinical correlation. Breast.

[15] Zhang L, Qin G, He Z, Chen W, Yang L (2016). The mammography and MRI manifestations of adenomyoepithelioma of the breast. Clin Radiol.

